# From soy to legumes: chemical diversity and sensory complexity of fermented sauces revealed by integrated analytical approaches

**DOI:** 10.3389/fnut.2026.1873081

**Published:** 2026-06-18

**Authors:** Margherita Modesti, Diana DeSantis, Perla Paoletti, Serena Ferri, Riccardo Riggi, Andrea Bellincontro

**Affiliations:** Department for Innovation in Biological, Agro-Food and Forest System, Tuscia University, Viterbo, Italy

**Keywords:** E-nose, legumes-based sauces, local production, NIR, soy sauces

## Abstract

**Introduction:**

The growing demand for sustainable and locally sourced food products has stimulated interest in alternative fermented condiments that can replace conventional soy sauce while maintaining comparable sensory and functional properties.

**Methods:**

In this study, commercial soy sauces and artisanal legume-based fermented sauces produced using traditional shoyu techniques were comparatively evaluated through an integrated analytical approach. Six samples, including three commercial soy sauces and three non-pasteurized sauces derived from peas, chickpeas, and lentils, were characterized in terms of physicochemical properties, volatile organic compounds, sensory profiles, consumer preference, and non-destructive measurements (electronic nose and near-infrared spectroscopy).

**Results and Discussion:**

Results demonstrated that raw material significantly influences both chemical composition and sensory perception. A total of 70 volatile compounds were identified. While soy-based sauces were characterized by a more defined volatile profile dominated by Maillard-derived compounds and volatile phenols, legume-based sauces exhibited a more complex volatile profile, including sulfur-containing metabolites and lipid oxidation products such as 1-octen-3-ol. This higher chemical diversity was reflected in increased sensory complexity, with descriptors such as dried mushroom, bacon, chocolate, clove, and Parmesan cheese showing the greatest discriminative power (*p* < 0.001). Consumer testing revealed significantly higher liking scores for legume-based sauces than for commercial soy sauces (*p* < 0.05), with chickpea-based sauce showing the highest acceptance. Non-destructive techniques effectively discriminated samples according to matrix composition. Electronic nose analysis explained 77.1% of the total variance in the first two principal components (PC1 = 59.3%, PC2 = 17.8%), whereas NIR spectroscopy explained 99.5% of total variance (PC1 = 87.2%, PC2 = 12.3%), clearly separating soy-based and legume-based products. The study demonstrates that locally sourced, soy-free fermented sauces obtained from legumes can achieve high chemical and sensory complexity, with strong consumer acceptance. These findings support the potential of such products as sustainable alternatives to conventional soy sauce within circular and low-impact food systems.

## 1 Introduction

Soy sauce is one of the most widely consumed fermented condiments worldwide, prized for its distinctive umami flavor and its long-standing role in East and Southeast Asian cuisines (1). Traditionally made by fermenting a mixture of soybeans and wheat with salt and water, soy sauce has evolved into a globally traded product used across diverse culinary cultures (2). Its global market was valued at approximately USD 56 billion in 2024 and is projected to reach USD 75–76 billion by 2030, underscoring both its culinary relevance and economic importance (3).

Despite its global appeal, conventional soy sauce production is associated with notable environmental challenges. The process relies heavily on soybeans and wheat, crops that are resource-intensive and often imported, particularly in non-soy-growing regions (4). Soybean cultivation alone accounts for over 77% of the water footprint in soy sauce production, and approximately 86% of total water consumption occurs upstream during agricultural cultivation rather than during fermentation (5). Industrial soy sauce production also generates between 6–9 m^3^ of high-strength effluent per ton of product, with elevated salinity and organic loads that pose significant wastewater treatment challenges (6). Additionally, the long fermentation time and energy demands contribute to a substantial environmental footprint (7). Furthermore, the global dependence on imported soy introduces vulnerabilities in supply chains and reduces the resilience of local food systems (8). It also contributes to deforestation and biodiversity loss in major soy-exporting countries (9, 10). These concerns underscore the need for more sustainable alternatives that maintain the sensory and functional qualities of soy sauce while minimizing environmental impact.

Although previous research has focused on optimizing soy-based fermentation products (11–13), relatively few studies have investigated alternative substrates that are both locally available and environmentally advantageous (14–16). Even fewer have explored the potential of upcycling non-commercial-grade agricultural products, such as visually imperfect or undersized legumes, that are typically excluded from retail supply chains despite being nutritionally adequate and microbiologically safe.

Soy sauce is produced through a two-stage fermentation process: the koji phase and the moromi phase (17). In the koji stage, soybeans and wheat are inoculated with *Aspergillus oryzae* or *A. sojae*, which release enzymes that degrade proteins and starches into peptides, amino acids, and sugars (2). This is followed by the moromi stage, in which the enzymatically treated mash is fermented for several months with halophilic yeasts (*Zygosaccharomyces rouxii*) and lactic acid bacteria (*Tetragenococcus halophilus*), generating hundreds of flavor compounds (2).

In addition to flavor, traditionally fermented foods like soy sauce have gained renewed attention for their potential health benefits (18). Fermentation can enhance nutritional profiles by increasing bioavailability of amino acids, generating bioactive peptides, and supporting gut health through the activity of beneficial microbes (18). Specific strains used in soy sauce fermentation, such as *Z. rouxii* and *T. halophilus*, have been shown to produce compounds with antioxidant and antimicrobial properties (11). Regular intake of fermented foods has been associated with improved digestive health, immune modulation, and overall increased microbiome health (19).

However, the health benefits of fermented condiments are often diminished by pasteurization, a common step in commercial soy sauce production (20). Pasteurization ensures shelf stability but also inactivates the live microbial populations and enzymes responsible for ongoing metabolic activity (11). As a result, many of the probiotic-like properties and enzymatic benefits are lost before consumption. This trade-off between microbial safety and functional value limits the nutritional potential of most commercial fermented condiments (20).

In response to the environmental and nutritional limitations of conventional soy sauce, this study investigates a real-world model of sustainable fermentation. The fermented sauces analyzed in this work are produced by a company located in central Italy driven by a zero-waste philosophy. The producer uses only raw materials that would otherwise be discarded, such as visually imperfect or undersized legumes, and applies the traditional fermentation process used for soy sauce making. However, instead of soy and wheat, the sauces are made from locally grown peas, lentils, chickpeas, and ancient cereals, all sourced locally from regional farms. No pasteurization is applied after fermentation, allowing the products to retain active microbial communities and enzymatic functions. This approach exemplifies a circular food system in which underutilized agricultural outputs are transformed into high-value, health-promoting condiments.

As such, the present study evaluates the chemical and aromatic composition as well as sensory characteristics of these alternative fermented sauces and compares them to conventional soy sauce. In addition to conventional laboratory assays, non-destructive analytical methods, including Near-Infrared (NIR) spectroscopy and electronic nose technology, were employed to explore the potential for predictive modeling of compositional and sensory attributes. Chemometric approaches are essential for correlating non-destructive measurements with references analyses using predictive models developed and validated through statistical approaches (21, 22). These tools offer rapid, solvent-free assessments and may support future applications in real-time quality control and process monitoring. By studying a working model that integrates traditional food processing with circular economy principles, this research aims to contribute to the broader discussion on resilient, low-impact food systems that valorize regional agrobiodiversity and reduce waste across the supply chain.

## 2 Materials and methods

### 2.1 Samples and production process

The study involved the chemical, sensory, and non-destructive characterization of six fermented sauce samples (Table 1). Three samples were commercial soy sauces commonly available in retail outlets, including a low-sodium variant. The remaining three samples were supplied by the local company Nesler Cibo Vivo (Strada Regionale 312 Castrense km, 12.250, 01011 Canino VT, Italy), which produces artisanal, non-pasteurized sauces by fermenting local legumes and cereals using traditional soy sauce fermentation techniques. For each sauce, three different bottles were analyzed as independent product replicates, and each bottle was subjected to three technical replicates for all destructive analyses.

**Table 1:** Samples summary and ingredients (as reported in the label).

Sample	Sauce type	Ingredients
Soy sauce 1	Commercial low salt soy sauce (pasteurized)	Water 49.5%, defatted soybeans 23%, wheat 18%, salt 7%, alcohol 2.4%, soybeans 0.1%
Soy sauce 2	Commercial soy sauce (pasteurized)	Water, defatted soybeans 17%, wheat, salt 16%, alcohol, soybeans 0.1%
Soy sauce 3	Commercial soy sauce (pasteurized)	Water, soybeans 18%, wheat 18%, salt 17%
Peas sauce	Artisanal pea- and wheat-based sauce (non-pasteurized)	Water, green peas 18%, wheat 18%, salt, whole sea salt 10%, *Aspergillus oryzae* spores
Chickpeas sauce	Artisanal chickpea- and wheat-based sauce (non-pasteurized)	Water, chickpeas 18%, wheat 18%, salt, whole sea salt 10%, *Aspergillus oryzae* spores
Lentils sauce	Artisanal chickpea- and wheat-based sauce (non-pasteurized, gluten free)	Water, black lentils 18%, spelt 18%, salt, whole sea salt 10%, *Aspergillus oryzae* spores

### 2.2 Chemical characterization

Standard analytical procedures were applied to all samples. A digital pH meter (Metrohm 713, Metrohm Ltd., Herisau, Switzerland) was used for pH measurement. Samples were diluted in distilled water for titration with 0.1 N NaOH (pH value of 8.3 was taken as the end point) and titratable acidity was expressed as % lactic acid (23). Total soluble solids were measured in diluted samples using a handheld analog refractometer and expressed as °Brix. Moisture content was determined by gravimetric drying at 105 °C (24). Each sample was analyzed in triplicate.

### 2.3 Volatile compound analysis (SPME-GC–MS)

Volatile organic compounds (VOCs) in the fermented sauces were analyzed by headspace solid-phase microextraction (HS-SPME) coupled with gas chromatography–mass spectrometry (GC–MS). For each sample, 2 g of fermented sauce were transferred into a 20 mL glass crimp vial, hermetically sealed with a silicone/PTFE septum, without the addition of sodium chloride. Samples were incubated at 40 °C for 15 min to allow headspace equilibration. VOCs were extracted at the same temperature using an SPME fiber (50/30 μm DVB/CAR/PDMS, 1 cm length; Supelco, Bellefonte, PA, USA) exposed to the headspace for an additional 15 min. After extraction, analytes were thermally desorbed from the fiber for 5 min in the GC injector set at 250 °C in splitless mode. GC–MS analysis was performed using a Clarus 680 Gas Chromatograph equipped with a split/splitless injector (PerkinElmer®, Waltham, MA, USA). Volatile compounds were separated on a fused silica capillary column (DB-Wax, 60 m × 0.32 mm i.d., 0.25 μm film thickness; Restek, Bellefonte, PA, USA), using helium as carrier gas at a constant flow rate of 1 mL·min^−1^. The GC was coupled to a Clarus 500 Mass Spectrometer (PerkinElmer®, Waltham, MA, USA). Compounds were identified by comparing mass spectra with those contained in the NIST mass library database. Only compounds with a spectral similarity index ≥ 75% were considered for identification (25). Each sample was analyzed in triplicate.

### 2.4 Sensory evaluation

A Rate-All-That-Apply (RATA) method was used for the descriptive sensory characterization of the six fermented sauces. Ten trained sensory judges (aged between 25 and 40 years), recruited from the Department of Agricultural and Forest Sciences (DIBAF) of the University of Tuscia (Viterbo, Italy), participated in the study. Panelists were selected, trained, and monitored according to ISO standards [8586**-**1, 8586**-**2 (26)]. The generation of sensory descriptors was conducted in individual panel booths of a sensory laboratory complying with ISO standard [8589, (27)]. Prior to the formal evaluation, two preliminary sessions were carried out to familiarize the assessors with the characteristics of the fermented sauce samples and to support descriptor generation. The development of the sensory lexicon followed the guidelines of ISO standard [13299 (28)]. During these sessions, samples were presented in monadic sequential order, and each panelist independently proposed descriptors considered representative of aroma, taste, mouthfeel, and aftertaste. All descriptors generated were collected and refined by eliminating redundant or irrelevant terms and merging synonyms. The final lexicon consisted of 45 sensory descriptors (Supplementary Table S1). The descriptive sensory evaluation was conducted by the same trained panel. Each judge evaluated the six samples, which were served in odorless plastic cups coded with three-digit random numbers. Samples were presented in randomized order under controlled environmental conditions. Judges rated the intensity of each selected descriptor using a seven-point scale (1 = very weak; 7 = very strong). Water was provided for palate cleansing between samples. In addition to the descriptive analysis, consumer preference was evaluated by 35 untrained consumers who reported regular soy sauce consumption (at least once per week). This inclusion criterion was adopted to ensure familiarity with the sensory characteristics of fermented soy-based condiments. Participants assessed only overall liking using a hedonic scale, without performing the descriptive RATA task. Consumer sessions were conducted under the same controlled sensory laboratory conditions. The study was conducted in accordance with the Declaration of Helsinki and was approved by the Ethics Committee of Tuscia University. Informed consent was obtained from all subjects involved in the sensory analysis.

### 2.5 Non-destructive analyses

#### 2.5.1 Electronic nose

The Electronic nose (E-nose) used in this study was designed and assembled at the University of Rome Tor Vergata and it is based on an array of 12 quartz microbalance (QMB) sensors. These sensors operate on the principle that small mass variations (Δm) occurring on the functionalized quartz surface generate proportional frequency shifts (Δf) in the oscillator circuit. Within the linear operating range, the frequency variation is directly proportional to the mass change. The QMB sensors consisted of AT-cut quartz crystals with a fundamental frequency of 20 MHz, corresponding to a mass resolution in the nanogram range. The sensors were functionalized with different metalloporphyrins, previously characterized for their sensitivity to VOCs. Each QMB was individually connected to an oscillator circuit, and a temperature-compensated quartz crystal was used as a reference to ensure a frequency resolution of 0.1 Hz. The system also included temperature and relative humidity sensors. Data acquisition and instrument control were managed through proprietary software developed in MATLAB (29). For sample measurement, 2 mL of sauce were transferred into 50 mL sealed glass vials equipped with a silicone septum and incubated at room temperature for 10 min to allow headspace equilibration. The equilibrated headspace was then extracted for 90 s using a filtered air flow and introduced into the sensor chamber of the E-nose. The aromatic enrichment of the headspace was evaluated by measuring the damping of the oscillation frequency of each sensor and comparing it to a baseline reference obtained using purified, odor-free, and humidity-free air. After each measurement, a pure air flow was applied for 300 s to clean the sensors and re-establish the baseline signal. The same air flow was used for cleaning between samples. Sensor responses were calculated as the shift in resonance frequency between the two steady-state conditions corresponding to exposure to pure air and to the sample headspace. The ensemble of sensor signals generated characteristic response patterns (fingerprints) encoding the overall composition of the volatile headspace (30). Each sample was analyzed in triplicate.

#### 2.5.2 NIR spectroscopy

A Luminar 5030 miniature, hand-held NIR analyzer (Brimrose Corporation, Baltimore, MD, USA), based on the AOTF-NIR principle, was used for spectral detection. NIR spectra were collected in the 1,100–2,300 nm range with a 1 nm step. Fifteen spectra were acquired for each sample and subsequently averaged. The spectra acquired in transmittance (T) mode were subsequently transformed into absorbance (A) by performing the calculation A = log(1/T) using SNAP! 2.03 software (Brimrose).

### 2.6 Statistical analysis

Destructive analyses were performed in triplicate for three independent sample (3 × 3). Physicochemical data were statistically evaluated using one-way analysis of variance (ANOVA), followed by Tukey’s *post hoc* test for pairwise comparisons, with statistical significance set at *p* ≤ 0.05. VOCs selection for heatmap visualization was performed using a supervised multivariate approach. A PLS-DA model was built on autoscaled data, and Variable Importance in Projection (VIP) scores were calculated. Compounds with VIP > 1 were selected as discriminant markers and used for heatmap construction. Bidirectional hierarchical clustering was then performed using Euclidean distance and complete linkage as clustering criteria. Based on these results, a heatmap was generated to visualize concentration patterns and clustering behavior among the fermented sauces. NIR spectra were mean-centered prior to multivariate analysis. Pre-treated NIR data and mean-centered E-nose datasets were organized into two distinct matrices for principal component analysis (PCA) with venetian blind cross-validation (blind thickness = 1). Sensory data were analyzed using PCA to evaluate the discriminative power of the selected sensory attributes in describing the profiles of the fermented sauces. To determine significant differences among samples based on intensity scores assigned by trained judges, a product characterization test was applied. For each descriptor, an ANOVA model was used to assess whether the assigned scores significantly differed among products. The model included both “product” and “judge” effects for each determination. The resulting model coefficients were examined for each product-descriptor combination to identify the most characterizing attributes contributing to product discrimination. Consumer preference data were analyzed using the non-parametric Kruskal-Wallis test, suitable for evaluating variance in datasets that do not follow a normal distribution. This method relies on rank transformation to normalize variability and is particularly appropriate for consumer data characterized by high inter-individual variability due to subjective use of hedonic scales. An exploratory analysis was performed to investigate potential associations between VOCs and sensory descriptors. VOC data obtained by GC–MS were autoscaled prior to multivariate analysis. Sensory descriptors not directly associated with volatile composition (namely color intensity, density, viscosity, astringent, bitter, acidity, salty) were excluded from the analysis. Mean sensory intensity scores obtained from the trained panel were used for analyses. Associations between VOCs and sensory descriptors were explored using Spearman’s rank correlation coefficients. Correlation matrices were visualized through hierarchical clustering heatmaps. All analyses were performed using XL-Stat (Lumivero, Denver, CO, USA), PLS Toolbox (Eigenvector Research, Inc., Manson, WA, USA), MATLAB R2013A (MathWorks, Natick, MA, USA), and RStudio (R version 4.5.2, 2025 R Foundation for Statistical Computing, Vienna, Austria).

## 3 Results and discussion

### 3.1 Chemical characterization

Table 2 reports titratable acidity, pH, TSS, and salt content of the six fermented sauces. Titratable acidity and pH are traditionally used to assess both quality and microbiological safety of fermented condiments. In Japan, soy sauce (shoyu) quality is defined by the Japanese Agricultural Standard (JAS), which classifies products into quality grades (31). Although JAS does not establish fixed limits for pH and titratable acidity, these parameters are intrinsic characteristics of fermented soy sauce and are strictly controlled by producers. Naturally fermented commercial soy sauces typically exhibit an acidic pH ranging from 4.6 to 5.2 (32). However, pH values may vary depending on fermentation type and duration. Studies on Japanese Koikuchi and Tamari soy sauces report slightly higher pH values (4.7–4.8), attributed to differences in microbial flora and enzymatic activity during fermentation (33). The acidity of soy sauce results from the formation of non-volatile organic acids, including malic, fumaric, pyroglutamic, lactic, citric, and succinic acids, as well as volatile acids such as acetic and butyric acid (34). Lactic acid and pyroglutamic acid are considered the primary contributors to sour taste perception (34). In the present study, titratable acidity ranged from 1.66 to 2.43 g/100 mL, in agreement with literature values (1.6–2.4 g/100 mL) reported for fermented soy sauces (35). Interestingly, the three soy-free fermented sauces (Peas, Chickpeas and Lentils sauces) exhibited slightly lower pH values compared to commercial soy sauces. This difference is directly linked to product safety requirements. Unlike commercial soy sauces, the artisanal legume-based sauces are not pasteurized and therefore must maintain a sufficiently low pH (< 4.6) to inhibit pathogenic microbial growth. Although total acidity generally correlates with lower pH values, soy sauce contains multiple weak organic acids, which buffer the system and prevent drastic pH decreases despite increases in total acidity (36).

**Table 2:** Titratable acidity (g lactic acid/100 mL), pH, total soluble solids (°Brix), salt content (%) as reported in the label, dry matter (%) and moisture content (%) in commercial soy sauces (soy sauce 1, 2 and 3) and artisanal legume-based sauces (peas sauce, chickpeas sauce, lentils sauce).

Sample	TA (lactic acid g/100 mL)	pH	TSS (° Brix)	Salt content (%)	Dry matter (%)	Moisture content (%)
Soy sauce 1	2.34 ± 0.12 a	4.95 ± 0.04 a	45.05 ± 0.07 a	7.80 ± 0.89 c	30.08 ± 0.18 a	74.45 ± 0.18 a
Soy sauce 2	2.43 ± 0.00 a	4.78 ± 0.05 a	40.50 ± 0.70 b	16.1 ± 0.22 a	28.14 ± 0.16 b	74.09 ± 0.15 a
Soy sauce 3	1.90 ± 0.06 b	4.74 ± 0.04 a	42.00 ± 1.41 ab	16.9 ± 0.45 a	27.18 ± 0.02 bc	73.25 ± 0.09 ab
Peas sauce	1.84 ± 0.12 b	4.44 ± 0.01 b	44.05 ± 0.07 ab	10 ± 0.35 b	26.75 ± 1.13 bc	72.81 ± 1.18 ab
Chickpeas sauce	1.71 ± 0.00 b	4.32 ± 0.02 c	40.50 ± 0.70 b	10 ± 0.12 b	25.9 ± 0.10 a	71.86 ± 0.17 b
Lentils sauce	1.66 ± 0.06 b	4.55 ± 0.01 b	42.00 ± 1.41 ab	10 ± 0.21 b	25.55 ± 0.15 a	69.91 ± 0.16 c

Values are the mean of 9 replicates ± SD. Different letters within columns represent statistical differences based on one-way ANOVA and Tukey post hoc test with *p* < 0.05.

TSS values ranged from 40.05 to 45.05 °Brix, confirming the high concentration of soluble solids typical of soy sauces and fermented condiments. According to the literature, TSS values can vary from 15 °Brix in non-concentrated soy sauces to over 50 °Brix in concentrated formulations (37). °Brix reflects the concentration of total soluble solids, including sugars, free amino acids, peptides, organic acids, and salts, and therefore represents a key indicator of compositional quality (36). The low-salt soy sauce and the Peas-based sample showed the highest TSS values (45.05 and 44.05%), possibly associated with higher levels of reducing sugars and free amino acids. Conversely, Commercial soy sauce 2 and Chickpeas-based sauce presented the lowest values (40.50 °Brix), suggesting either more diluted formulations or differences in fermentation dynamics. Soluble solids contribute not only to taste, particularly sweetness and umami, but also to aromatic properties by influencing volatile compound retention and sensory persistence (38).

Traditional naturally fermented soy sauce typically contains between 18–20% salt (w/v) (39). However, growing health awareness and recommendations to reduce sodium intake have led to the development of reduced-salt formulations, achieving approximately 40–50% sodium reduction compared to traditional products. Salt reduction strategies include post-fermentation desalination, partial substitution of sodium with potassium or calcium salts, or fermentation under reduced-salt conditions (39). In the present study, the declared salt content ranged from 7.8% in the commercial low-salt soy sauce to 16.9% in the commercial Soy sauce 3. The commercial Soy sauce 2 and 3 showed salt percentages close to traditional formulations (16–17%), whereas the three legume-based sauces contained 10% salt, reflecting an intermediate formulation between traditional and reduced-salt products.

Moisture content ranged from 69.92 to 74.45%, values consistent with high-moisture fermented sauces. Soy sauces typically contain high water percentages, forming the continuous phase that solubilizes taste-active compounds. Previous studies report moisture contents around 59–60% for soy sauce residues, while dry matter can vary widely (13.6–77.2%) depending on production methods (40). In the present study, Commercial soy sauce 1 (low-salt) and Chickpeas-based sauce showed the highest moisture values (74.09 and 74.45%), corresponding to the lowest dry matter content. Commercial soy sauce 2 and Lentils-based sauces showed intermediate values, whereas Commercial soy sauce 3 and peas-based sauce exhibited relatively lower moisture content. Moisture and dry matter influence technological, sensory, and microbiological properties. Water affects aroma solubility, viscosity, and taste intensity. Dry matter includes carbohydrates, proteins, NaCl, organic acids, and fermentation-derived compounds that contribute to aromatic density, persistence, and product stability. Differences among samples likely reflect raw material composition, fermentation duration, and processing methods.

### 3.2 Volatile profile

The volatile profile of the six sauces was investigated by SPME GC–MS. Overall, 70 volatile compounds were detected (match ≥ 75%, Supplementary Table S2), belonging to several chemical classes typically associated with fermented condiments, including alcohols, esters, aldehydes, ketones, acids, furans/furanones, volatile phenols, sulfur- and nitrogen-containing compounds.

Alcohols represented one of the most abundant chemical families detected in the fermented sauces. Consistent with previous literature on soy sauce fermentation (41), ethanol was among the dominant alcohols identified, confirming its role as the primary product of sugar metabolism during fermentation. Ethanol formation is driven by yeast activity and represents a key contributor to the characteristic alcoholic and fermentative aroma of soy-based condiments. In addition to ethanol, several higher alcohols were detected, including 1-Propanol, 1-Propanol-2-methyl, 1-Butanol, 3-methyl-1-butanol, 1-Octen-3-ol, furfuryl alcohol, benzyl alcohol, phenylethyl alcohol. These compounds are commonly reported in fermented soy sauces (42) and arise from amino acid catabolism and lipid oxidation pathways. For instance, 1-Propanol 2-methyl contributes faint bitter notes (43), whereas benzyl alcohol provides almond-like aromas (13). Of particular interest, 1-octen-3-ol, often described as “mushroom-like,” is typically formed via lipid oxidation during fermentation (7).

Esters are key contributors to fruity and sweet aromatic notes in fermented sauces. In agreement with literature (44, 45), characteristic esters such as lactic acid ethyl ester, benzene acetic acid ethyl ester, acetic acid phenethyl ester, butanoic acid ethyl ester, ethyl caproate and ethyl caprylate were detected. Most esters are produced through microbial esterification reactions between alcohols and organic acids during the later stages of fermentation (46). Their formation often coincides with increased sensory complexity, contributing fruity, floral, and sweet nuances that soften sharp acidic or alcoholic notes. In soy-based sauces, ester formation is typically associated with prolonged fermentation and balanced yeast activity. In legume-based sauces, variations in ester abundance may reflect differences in available precursors (free fatty acids, higher alcohols) and lipase-mediated reactions, particularly under long-term maturation conditions.

Aldehydes constitute another important class of volatile compounds in fermented condiments. Compounds such as benzaldehyde, 2-hexanal, and 2-furfural were detected and are consistent with previous soy sauce fermentation studies (7). Short-chain and branched aldehydes are typically generated through amino acid degradation (via Strecker degradation) and Maillard-type reactions (46). Benzaldehyde contributes burnt sugar or caramel-like notes, while hexanal is associated with green and fatty notes. Differences in aldehyde profiles among samples may reflect variations in protein composition, lipid oxidation, and thermal history. Legume-based sauces, characterized by different amino acid patterns compared to soy, may generate distinct aldehyde signatures, influencing perceived sweetness, nuttiness, or oxidative notes.

Volatile phenols, such as guaiacol, were identified and are known contributors to smoky and toasted aromas in soy sauce (47). These compounds are typically formed through microbial metabolism of ferulic acid and related phenolic precursors. Their presence is particularly relevant in sauces aged in wooden barrels or undergoing long fermentation, where enzymatic and microbial transformations promote phenolic release.

Ketones and furan derivatives are characteristic contributors to caramel-like and sweet sauce aromas. Among them, 4-hydroxy-2-methyl-5-ethyl-3(2H)-furanone (HEMF) is recognized as one of the key aroma-active compounds in soy sauce, conferring caramel, creamy, and sauce-like notes. HEMF formation is mainly associated with carbohydrate metabolism and Maillard-related reactions (48). Its detection supports the presence of complex carbohydrate-protein interactions during fermentation and maturation.

Although both commercial soy sauces and legume-based sauces shared many volatile families typical of fermented condiments, relative differences were observed across samples. These differences likely arise from distinct amino acid profiles (soy vs. peas, chickpeas and lentils), variations in mineral composition as well as differences in lipid content and oxidation pathways. In addition, processing-related factors, including artisanal versus industrial production, pasteurization status, formulation variability, salt concentration, and manufacturer-specific fermentation conditions, may also have contributed to the observed volatile differentiation among samples.

**Figure 1:**
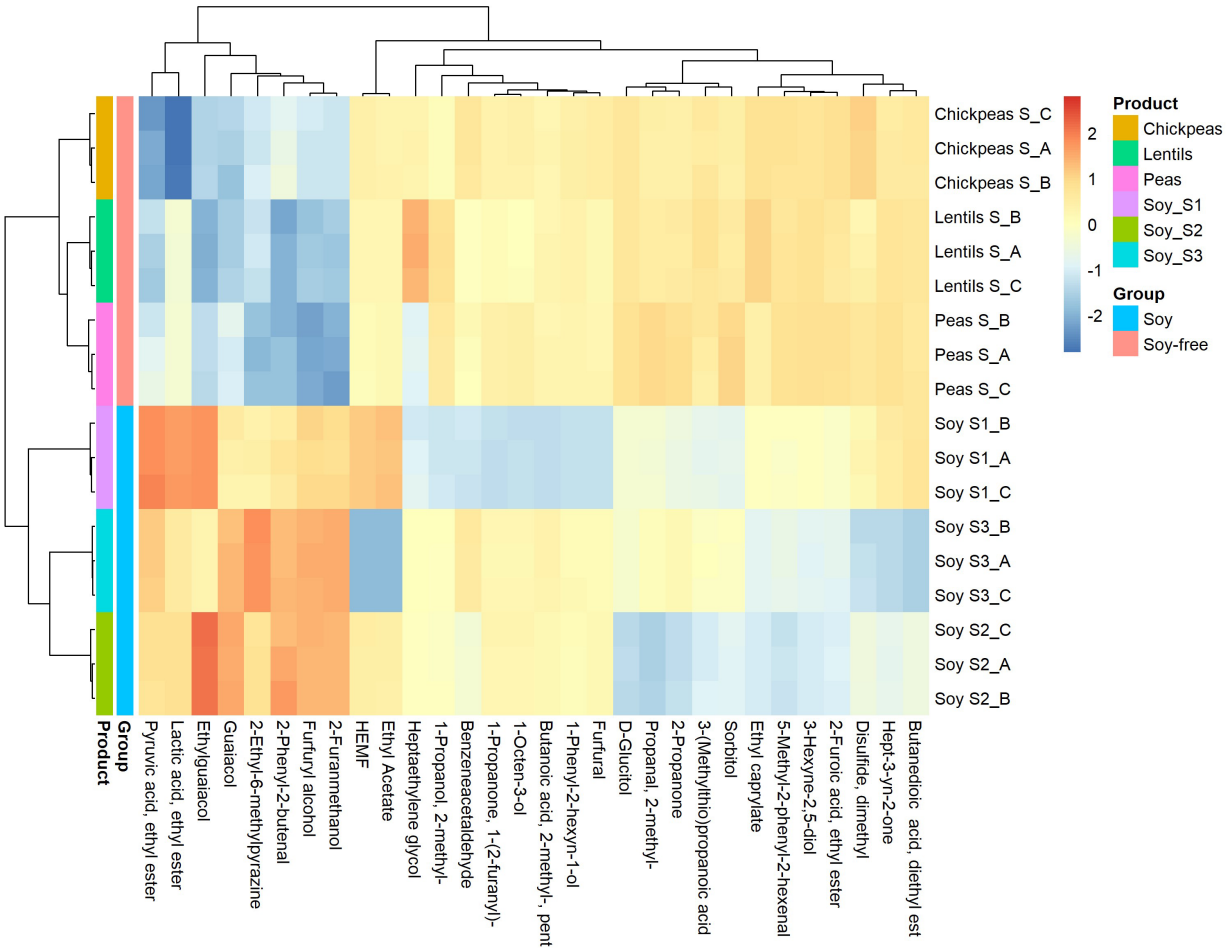
Heatmap of the most discriminant VOCs selected based on VIP scores derived from the PLS-DA model. Data are shown as row-wise Z-score normalized values of log-transformed peak areas. Red indicates higher relative abundance, whereas blue indicates lower relative abundance relative to the mean value of each compound across all samples. Hierarchical clustering was performed using Euclidean distance and complete linkage.

As previously specified, a preliminary PLS-DA (Supplementary Figure S1) was carried out on autoscaled data to identify the VIP (Supplementary Table S3) to be used for heatmap production. The heatmap based on VIP-selected volatile compounds (Figure 1) revealed a clear structuring of samples primarily according to raw material origin. Hierarchical clustering separated commercial soy sauces from legume-based fermented sauces, indicating that substrate composition, as well as commercial/artisanal production, exerts a dominant influence on volatile development, even under comparable shoyu-style fermentation conditions. The primary separation observed in the dendrogram reflects systematic differences in the relative abundance of key chemical families. Commercial soy sauces were characterized by comparatively higher levels of short-chain esters (e.g., ethyl acetate), benzaldehyde, and furan derivatives, compounds previously associated with fruity, almond-like, and caramelized notes (49). These volatiles are commonly generated through yeast-driven esterification and amino acid degradation in soybean-wheat matrices, where fermentable sugars and specific aromatic precursors are readily available. Additionally, the elevated levels of guaiacol and ethyl guaiacol in commercial soy sauces, known to have low odor thresholds, might impact smoky, spicy, and clove-like sensory attributes as already demonstrated (50).

On the other hand, the legume-based sauces exhibited elevated relative intensities of sulfur-containing compounds (e.g., 3-methylthio-propanoic acid, dimethyl disulfide), 1-octen-3-ol, and selected pyrazines. In traditional soy sauce, glutamate-driven umami perception is central. In soy-free systems, enhanced production of sulfur volatiles and roasted heterocycles may contribute to perceived depth and savory complexity, as previously reported (51), offsetting the absence of soy-derived precursors. The enrichment in sulfur-derived metabolites suggests alternative amino acid catabolic pathways driven by the specific protein composition of peas, chickpeas, and lentils. These legumes differ significantly from soybean in terms of methionine- and cysteine-derived precursor availability (52), which may enhance the formation of thio-compounds often associated with savory and fermented notes. Pyrazines, typically associated with roasted and nutty notes, were present across matrices but exhibited differential relative intensities (53). Their formation is linked to Maillard-type reactions and amino acid degradation; thus, differences in protein composition likely underlie the observed variation. The prominence of 1-octen-3-ol in legume-based samples is noteworthy, as this compound has frequently been associated with mushroom-like and earthy notes in fermented and plant-based matrices (54).

Clearly, substrate composition drives volatile differentiation between matrices. Soy-based sauces are characterized by a smaller number of dominant marker compounds with relatively higher relative intensities. In contrast, legume-based sauces exhibit a greater number of volatile compounds distributed across multiple chemical classes, indicating higher overall chemical diversity.

Within the soy-based cluster, Commercial soy sauce 2 and 3 exhibited a high degree of similarity, consistent with comparable salt concentrations. Their volatile profiles showed balanced distributions of esters and aldehydes. The low-salt soy sauce displayed partial separation from the other commercial products. Reduced salt concentration (7.8% vs. 16–17%) likely modifies osmotic stress conditions, influencing yeast activity and ester formation dynamics. Moreover, salt concentration is known to regulate microbial succession and enzymatic activity during fermentation (55) as well as aroma volatilization.

Among soy-free sauces, the clustering of Chickpeas and lentils-based sauces implies similar precursor pools and fermentative pathways, possibly related to comparable protein structures and mineral composition. In contrast, the distinct positioning of Peas-based sauce may reflect differences in carbohydrate composition and available reducing sugars, which can modulate Maillard-related and heterocyclic compound formation. Such differentiation highlights that legume identity itself becomes a key driver of volatile evolution, beyond the shared fermentation protocol.

### 3.3 Sensory analysis

#### 3.3.1 Sensory characterization by trained panel

The sensory data were analyzed using a product characterization approach aimed at identifying the descriptors most effective in discriminating among the six fermented sauces. For each sensory attribute, a two-way ANOVA model was applied, considering both “product” and “judge” effects. This model allowed the evaluation of whether the intensity scores assigned by the trained panel significantly differentiated the products, returning test statistics and associated *p*-values for each product–descriptor combination. As reported in Table 3, the most discriminating attributes included dried mushroom, bacon, chocolate, bitter, clove, and Parmesan (all *p* < 0.001), indicating that roasted, fermentative, and savory dimensions played a major role in product differentiation. Conversely, attributes such as viscosity, color intensity, and umami showed limited discriminative power and did not significantly differentiate the samples, suggesting that these attributes were perceived similarly across products or were not primary drivers of sensory variability.

**Table 3:** Discriminative power of sensory descriptors obtained from the RATA analysis, based on ANOVA *F*-values and associated *p*-values.

Descriptors	Test values	*p*-values
Dried mushroom	7.033	0.000
Bacon	6.627	0.000
Chocolate	5.868	0.000
Bitter	5.542	0.000
Clove	5.104	0.000
Parmesan cheese	4.521	0.000
Pungent	4.332	0.000
Licorice	4.291	0.000
Salty	4.024	0.000
Sweet	4.008	0.000
Overall preference	3.841	0.000
Marsala wine	3.799	0.000
Toasted	3.637	0.000
Sour	3.478	0.000
Smoky	3.319	0.000
Density	3.267	0.001
Meat	3.097	0.001
Coffee	2.401	0.008
Astringent	1.214	0.112
Color intensity	0.708	0.239
Viscous	0.426	0.335
Umami	0.228	0.410

Table 4 summarizes the adjusted means of each descriptor across the six samples. Chickpeas-based sauce emerged as the most sensorially complex matrix among all samples, exhibiting positive associations (blue cells) with a several descriptors. Its profile is primarily defined by bacon, smoked, and meat notes, conferring a distinctly savory and proteinaceous character. These attributes are chemically coherent with the elevated levels of sulfur-containing compounds detected in the volatile analysis, which are known contributors to meaty and savory aroma impressions (56). The strong association with dried mushroom aligns with the higher relative abundance of 1-octen-3-ol, a lipid oxidation-derived alcohol widely recognized for its mushroom-like aroma (54). Likewise, the presence of Parmesan and Marsala descriptors suggests fermentative and mildly oxidative nuances, consistent with the complex sulfur and heterocyclic volatile pattern observed in this sample.

**Table 4:** Adjusted mean values of sensory descriptors across the six fermented sauces.

Descriptor	Chickpeas-based sauce	Peas-based sauce	Commercial soy sauce 3	Commercial soy sauce 1	Lentils-based sauce	Commercial soy sauce 2
Bacon	4.000	2.600	0.000	1.600	1.000	0.000
Smoky	3.200	2.400	3.000	3.000	2.200	1.400
Parmesan cheese	5.000	2.800	1.800	1.600	2.400	2.200
Sweet	3.600	2.200	3.600	2.600	2.200	1.600
Density	4.200	3.800	3.000	2.300	2.864	2.800
Licorice	4.600	1.800	3.800	2.400	3.200	1.800
Meat	4.000	3.400	4.200	4.400	3.400	1.800
Dried mushroom	4.600	5.200	0.000	0.000	2.800	1.200
Marsala wine	4.800	4.200	2.000	1.800	4.400	2.600
Acidity	4.200	4.000	4.600	3.200	4.600	3.200
Chocolate	2.800	3.200	4.400	0.000	4.200	0.000
Coffee	3.600	3.000	4.800	2.800	3.297	3.400
Color intensity	4.538	4.800	4.200	5.200	4.800	4.600
Toasted	3.200	2.200	4.200	3.000	2.600	3.600
Viscosity	2.200	1.800	2.000	2.000	2.800	2.400
Astringent	1.800	2.800	2.200	2.400	2.600	2.200
Bitter	1.000	4.000	1.000	2.000	2.000	3.000
Umami	4.600	5.000	5.200	4.600	5.400	5.200
Clove	0.000	1.200	1.400	0.000	2.400	2.000
Salty	4.000	4.600	5.600	4.600	4.400	6.200
Pungent	2.400	2.600	3.200	2.400	3.400	4.600

Blue and red cells indicate positive and negative contributions, respectively, to the sensory profiles of the products.

A second cluster of descriptors defining Chickpeas-based sauce includes sweet, chocolate, coffee, toasted, and licorice. These attributes are chemically supported by the presence of pyrazines and furan derivatives, which are typical products of Maillard-type reactions and amino acid degradation during fermentation. In particular, heterocyclic compounds such as furfural and related derivatives contribute to roasted and caramelized notes, while pyrazines are associated with coffee-like and toasted perceptions. The positive association with density suggests a fuller tactile perception, reinforcing the idea of a structured and concentrated matrix. The contribution of acidity enhances sensory vibrancy and supports overall complexity.

Peas-based sauce displays a partially overlapping but less intense sensory configuration compared to Chickpeas-based sauce. Positive association with bacon, Parmesan, dried mushroom, Marsala, acidity, chocolate, and color intensity indicate a structured but more moderated aromatic profile. The presence of dried mushroom again corresponds to 1-octen-3-ol, though at relatively lower intensities than in Chickpeas-based sauce. The chocolate and roasted notes are supported by pyrazines and furan compounds, albeit in a less dominant manner. Unlike Chickpeas-based sauce, Peas-based sauce shows weaker associations with smoked and meat descriptors. Interestingly, descriptors such as astringent, bitter, umami, and clove, more typical of commercial soy sauces, play a more central role in Peas-based sauce. This suggests that Peas-based sauce occupies a transitional sensory space between legume-based fermentations and traditional soy matrices. Lentils-based sauce presents an intermediate sensory identity. It is positively associated with licorice, marsala, acidity, dried mushroom, chocolate, umami, and clove. The mushroom descriptor again reflects the contribution of 1-octen-3-ol, while chocolate and roasted notes are supported by heterocyclic Maillard-derived compounds. Positive association with viscosity, pungency, and astringency indicate a relevant tactile dimension. However, compared to Chickpeas-based sauce, Lentils sauce exhibits lower sensory intensity, suggesting a more balanced but less intense aromatic complexity. Collectively, the three legume-based sauces share a common sensory backbone, dried mushroom, Marsala, acidity, and chocolate, which mirrors their broader and more chemically diverse volatile composition.

On the other hand, Commercial soy sauce 3 shows a complex but directionally distinct sensory profile. Positive associations include smoked, sweet, licorice, meat, acidity, chocolate, coffee, toasted, umami, clove, salty, and pungent descriptors. This configuration is consistent with its volatile composition, characterized by elevated levels of furan derivatives (including furfural), and phenolic compounds such as guaiacol and ethyl guaiacol. These compounds contribute roasted, smoky, and spicy notes typical of traditionally fermented soy sauces. The umami descriptor reflects proteolytic degradation products inherent to soybean fermentation and represents a central sensory feature of this matrix. Negative association with bacon, Parmesan, dried mushroom, and Marsala highlight a fundamental structural difference from legume-based sauces. Commercial soy sauce 3 identity is not characterized by cheese-like or broth-like fermentative nuances but rather on saltiness, roasted character, and protein-derived aromatic intensity. Commercial soy sauce 2 is primarily defined by toasted, viscous, bitter, umami, clove, salty, and pungent attributes. The profile appears more focused and less diversified than that of Chickpeas-based sauce or Commercial soy sauce 3, reflecting a narrower volatile distribution dominated by specific Maillard and phenolic markers. Soy Low Salt (Commercial soy sauce 1) exhibits the least complex sensory profile. Positive correlations are limited to bacon, smoked, meat, color intensity, and astringency. This restricted sensory profile is consistent with the volatile analysis, which identified this sample as having the lowest overall volatile intensity. The reduction of key esters, heterocyclic compounds, and phenolic derivatives translates into a less differentiated sensory identity.

#### 3.3.2 Consumers preferences

**Figure 2:**
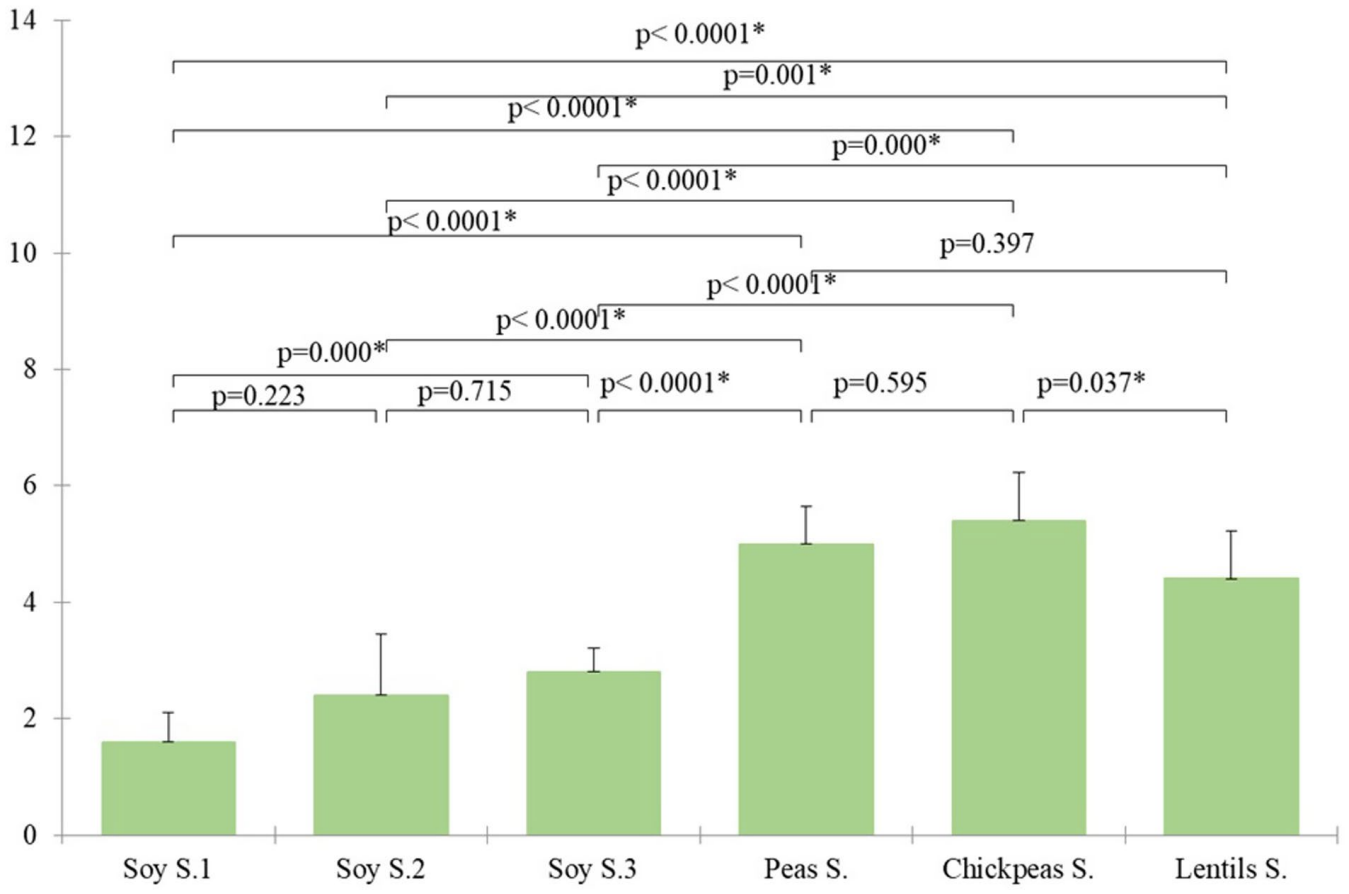
Consumer preference scores (0–7 hedonic scale) for the six fermented sauces. Bars represent mean values (*n* = 35) ± standard deviation. Asterisks (*) indicate statistically significant differences among samples (Kruskal–Wallis test, *p* < 0.05).

To assess consumer acceptability across the different fermented sauces, hedonic evaluations were collected using a 0–7 preference scale through an affective test (Figure 2). Preference analysis revealed a clear tendency of the consumer panel to favor legume-based fermented sauces over commercial soy sauces (*p* < 0.05). Specifically, within the consumer panel considered in this study, legume-based sauces obtained significantly higher liking scores compared to the three commercial soy sauces. Among all samples, Chickpeas-sauce achieved the highest mean preference score, consistent with its highly complex sensory profile observed in in volatile and sensory profiling. Chickpeas-sauce was characterized by strong associations with bacon, smoked, meat, Parmesan, dried mushroom, Marsala, sweet, chocolate, toasted, coffee, licorice, and higher density. The convergence of smoked, meaty, and sweet–roasted attributes appears to have positively influenced hedonic perception. This suggests that a complex yet balanced aromatic structure, supported by both fermentative and Maillard-derived notes, is perceived as particularly pleasant by consumers. Peas-based sauce, whose descriptive profile partially overlapped with chickpeas sauce (bacon, Parmesan, dried mushroom, Marsala, sour, chocolate, color intensity, and density) but with reduced sweet and smoky intensity, showed intermediate preference scores. Lentils-based sauce exhibited moderate liking scores. Its descriptive profile, associated with licorice, marsala, sour, dried mushroom, chocolate, umami, clove, and textural attributes such as viscosity, pungency, and astringency, indicates a multifaceted but less intense aromatic structure. While consumers appear to appreciate the sweet–roasted and umami dimensions, the lower intensity and more heterogeneous balance of attributes may have reduced its hedonic impact relative to Chickpeas-sauce. In contrast, the three commercial soy sauces generally received lower preference scores. This trend aligns with their descriptive profiles, which were dominated by traditional soy sauce attributes such as umami, salty, pungent, toasted, astringent, bitter, and clove. Although these descriptors define the canonical identity of soy sauce, they were less positively associated with consumer liking in the present study compared to smoked, meaty, and sweet–roasted notes. In particular, the low-salt soy sauce, previously identified as the least complex matrix in both volatile and descriptive analyses, obtained the lowest liking scores. Its reduced aromatic intensity and limited number of positively associated descriptors support the hypothesis that sensory richness and multidimensionality contribute positively to consumer acceptability.

#### 3.3.3 Relationships between volatile composition and sensory perception

**Figure 3:**
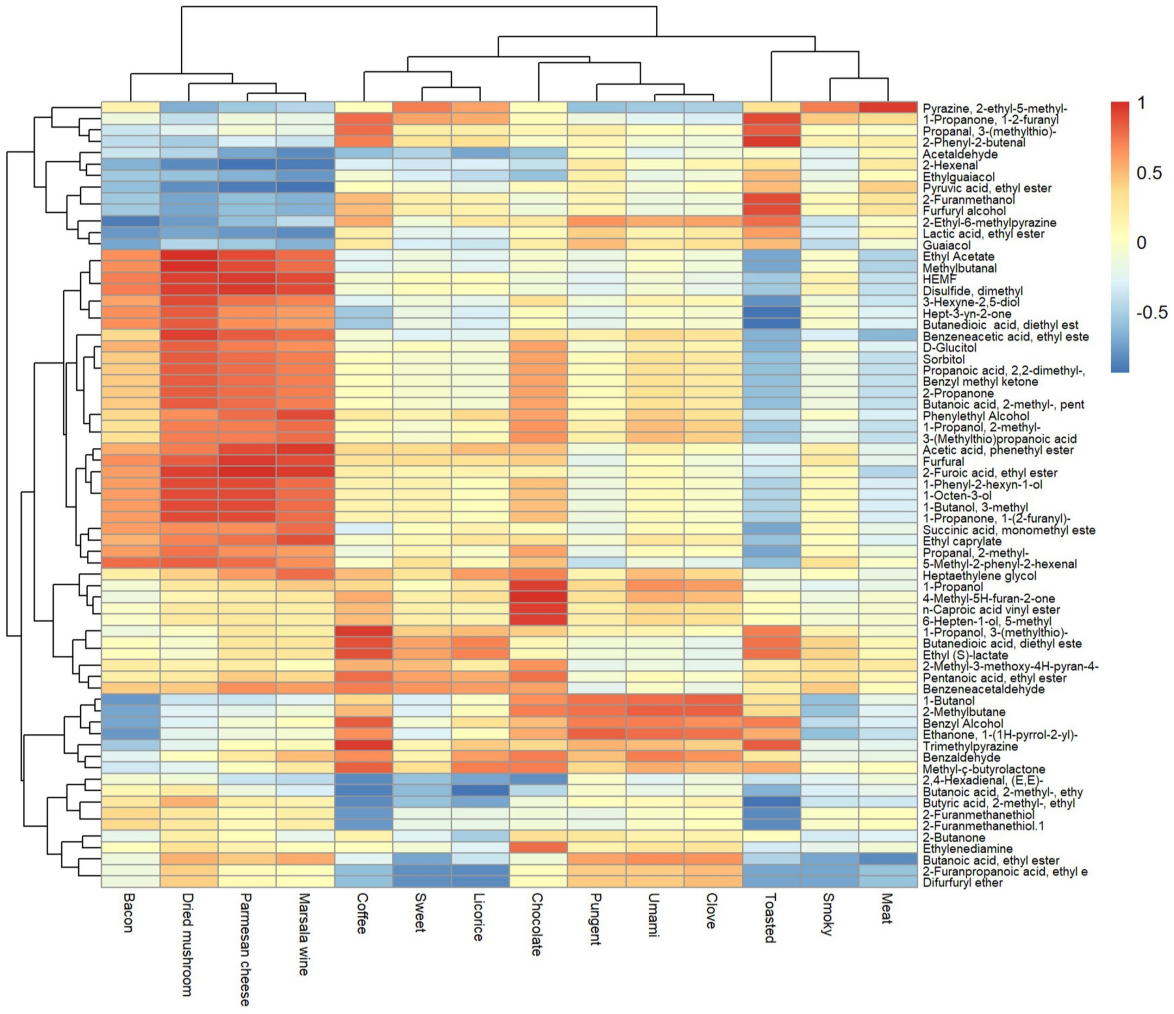
Exploratory Spearman correlation between VOCs and sensory descriptors.

To further explore the relationship between chemical composition and sensory perception, an exploratory association analysis was performed between VOC relative abundances and sensory attributes (Figure 3). Since VOC data were based on relative abundances rather than absolute quantification, the observed relationships should be interpreted as association patterns rather than direct evidence of sensory contribution.

Hierarchical clustering of the correlation matrix revealed distinct groups of sensory descriptors characterized by similar association patterns with the volatile dataset. A first cluster included dried mushroom, Parmesan cheese, Marsala wine, and bacon descriptors, which exhibited strong positive associations with several fermentation-derived compounds, including 1-octen-3-ol, sulfur-containing metabolites, esters, and Maillard-related compounds. In particular, 1-octen-3-ol showed a clear positive association with this sensory domain, consistent with its frequent description as a mushroom-like and earthy odorant in fermented foods (54). Sulfur-containing compounds, such as 3-methylthiol propanoic acid and dimethyl disulfide, were also positively associated with these descriptors. These metabolites originate from sulfur amino acid degradation and have previously been linked to savory, fermented, and mature aromatic notes. Additional positive associations were observed for phenylethyl alcohol, acetic acid phenethyl ester, ethyl caprylate, furfural, and HEMF, suggesting that esters and Maillard-derived compounds may contribute to the perceived aromatic complexity of these products (48).

A second cluster grouped coffee, chocolate, sweet, and licorice descriptors. These attributes were positively associated with several compounds generated through fermentation and Maillard-related pathways, including pyrazine derivatives, furfuryl alcohol, 2-furanmethanol and benzaldehyde. Pyrazines are widely recognized contributors to roasted, nutty, and coffee-like aromas, while furans and furanones are commonly associated with sweet, caramel-like, and cocoa-like sensory perceptions (49).

A third sensory domain comprised toasted, smoky, clove, pungent, and umami descriptors. These attributes were associated with volatile phenols, including guaiacol and ethylguaiacol, together with selected pyrazines and fermentation-derived esters such as ethyl lactate and pyruvic acid ethyl ester. Guaiacol and ethylguaiacol are well-known contributors to smoky, spicy, and clove-like aromatic perceptions and are frequently reported among the characteristic aroma compounds of traditionally fermented soy sauces (47). The association of these compounds with toasted and smoky descriptors is therefore consistent with the sensory profiles observed for the commercial soy sauces. Interestingly, this cluster displayed an association pattern distinct from that observed for the mushroom–Parmesan–Marsala group, suggesting the presence of two different aromatic signatures within the fermented sauces investigated.

### 3.4 Non destructive evaluation

#### 3.4.1 Aromatic fingerprint through E-nose

**Figure 4:**
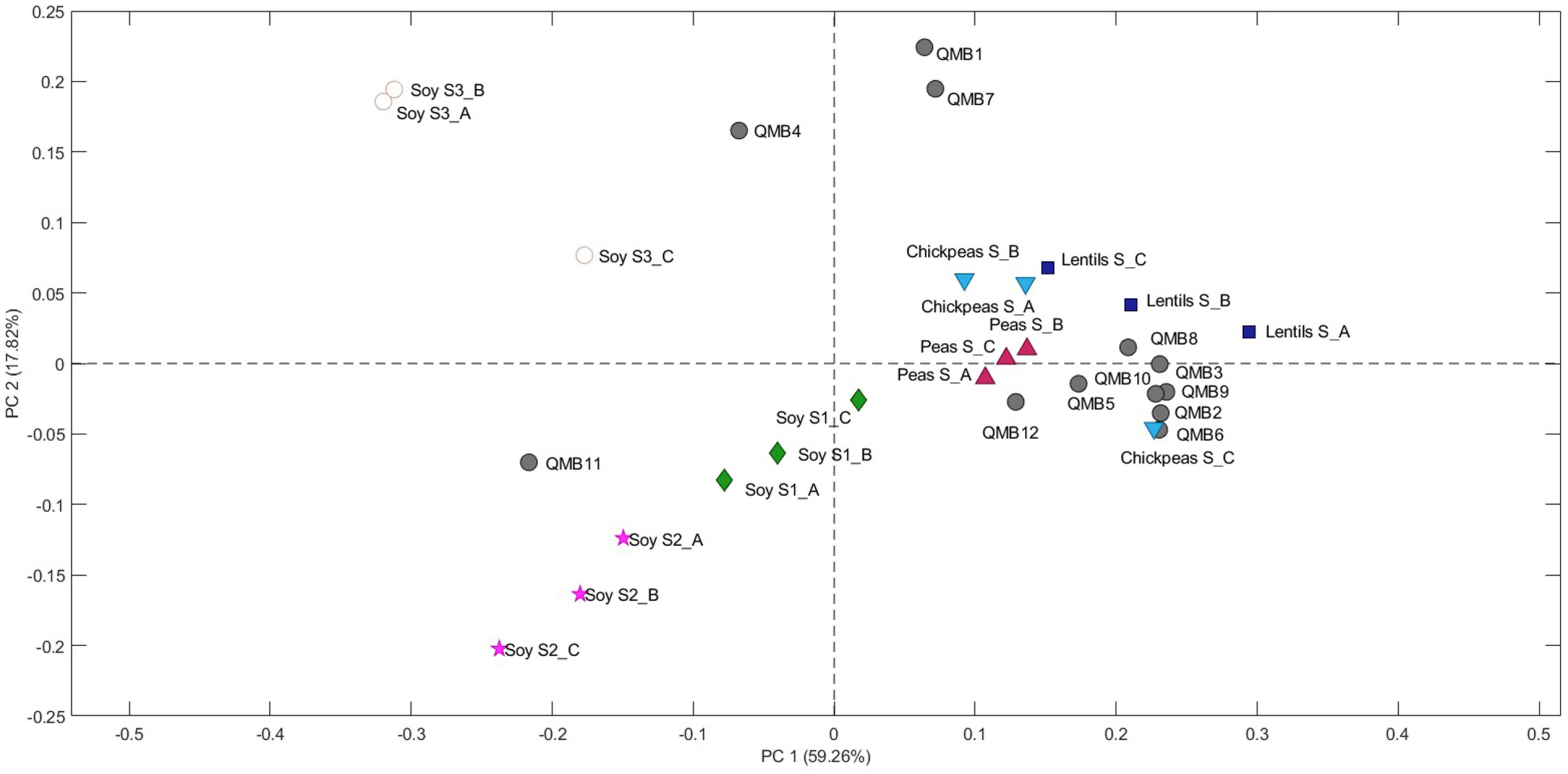
PCA biplot (PC1 vs. PC2) obtained from the E-nose responses of commercial soy sauces and legume-based fermented condiments.

The application of E-nose systems in soy sauce analysis has shown considerable potential, although their use remains relatively limited in industrial practice (57). Early studies (58) emphasized the challenges associated with directly correlating sensor responses to specific sensory attributes, mainly due to the intrinsic selectivity and cross-sensitivity of sensor arrays. To date, most research efforts have focused on differentiating among commercial soy sauce varieties rather than exploring broader matrix diversification (59). More recent studies have demonstrated that E-nose systems, particularly when integrated with complementary analytical techniques such as GC–MS and multivariate statistical analysis, can effectively discriminate between different soy sauce types and identify the key volatile patterns responsible for their differentiation (60, 61). In this context, the E-nose can be used as a rapid and non-destructive tool capable of capturing the global aromatic fingerprint of fermented matrices. In the present study, the first principal component (PC1), explaining 59.26% of the variance, showed a marked separation between soy-based and soy-free sauces, indicating that the overall volatile fingerprint captured by the sensor array is strongly driven by the raw material. The second principal component (PC2), although explaining a lower proportion of variance (17.82%), captured intra-group variability (Figure 4). For instance, the broader dispersion observed for chickpeas-based sauces along PC2 agrees with their wider distribution of volatile compounds across different chemical classes and their strong sensory profile, characterized by fermentative, roasted, and pungent attributes. In contrast, the tighter clustering of pea-based samples reflects a more homogeneous volatile composition and a less intense sensory expression, as also indicated by their more moderate association with key discriminating descriptors.

A key feature of the biplot is the distribution of the QMB sensors, which are predominantly oriented toward the positive side of PC1 and closely associated with legume-based samples. This pattern indicates that these matrices activate a larger number of sensors simultaneously, suggesting a broader and more complex volatile fingerprint. In particular, QMB2, QMB3, QMB5, QMB6, QMB8, QMB9, and QMB10 were strongly associated with legume-based sauces. The involvement of such a wide range of sensors supports the presence of a more diverse set of volatile compounds, in agreement with GC–MS results and sensory analysis, which highlighted a higher chemical and sensory complexity for these samples. In contrast, soy-based sauces were associated with a much more limited number of sensors, mainly QMB1 and QMB11, each linked to specific samples. This restricted sensor engagement suggests a more defined and less complex volatile composition, dominated by a smaller number of key aroma-active compounds. This interpretation is also consistent with both GC–MS data, which indicated a more concentrated distribution of specific VOC classes, and sensory results, where soy sauces exhibited a more focused aromatic profile compared to the multidimensional sensory profile of legume-based sauces.

#### 3.4.2 NIR spectra

**Figure 5:**
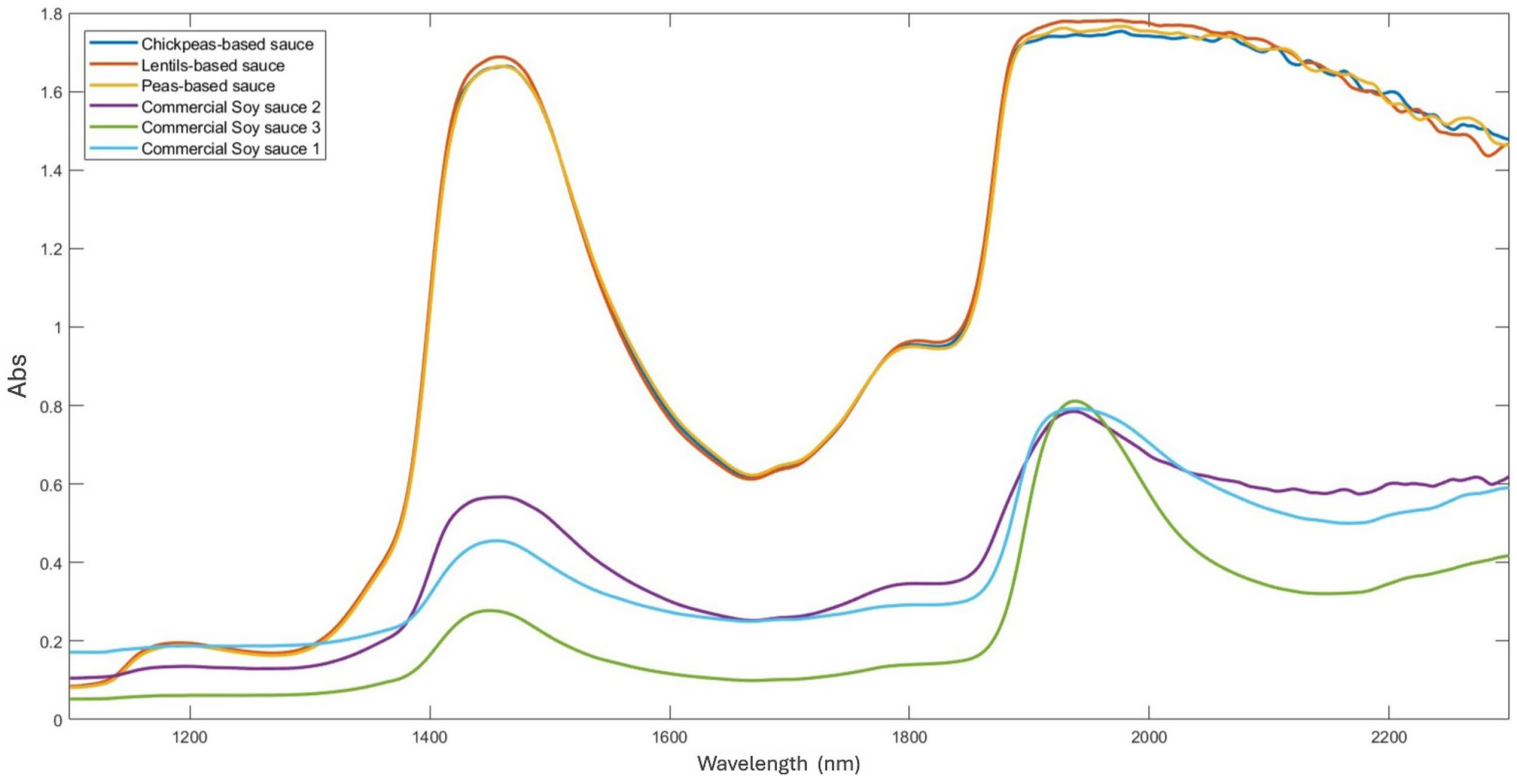
Mean NIR spectra (1100–2,300 nm) of the fermented sauces, averaged across replicates for each product class.

NIR spectroscopy is a widely applied, rapid, and non-destructive analytical technique requiring minimal sample preparation, which has already been used for soy sauce quality monitoring (62–65). The NIR region (780–2,500 nm) contains information related to C–H, O–H, N–H, and S–H bonds, which constitute the main structural components of organic molecules. Spectral features in this region arise from overtone and combination vibrational transitions associated with these functional groups (66). In this study, NIR measurements were integrated with conventional physicochemical analyses to provide complementary, non-destructive chemical characterization. Overall, soy-based sauces showed generally lower absorption signals. All samples exhibited prominent absorption bands around 1,450 nm and 1990 nm (Figure 5), attributed to the first overtone of free O-H stretching and O-H combination bands of water molecules, respectively (67). This observation is consistent with the high moisture content (69–75%) measured in the fermented sauces. Additional spectral differences were observed in the 1,100–1,300 nm region, particularly in legume-based condiments, likely associated with C-O and O-H functional groups of sugars, alcohols, and phenolic compounds (67). Moreover, a small absorption feature around 1800 nm was visible in legume-based samples, previously related to N-H vibrations of proteinaceous components (65). This may reflect differences in protein composition between soybean and legume-based matrices. For legume-based condiments, the highest signal was registered in the region located between 1,400–1,600, corresponding to O-H and N-H absorptions related to water-protein interactions. Additional contributions were observed around 2,200–2,300 nm, typically associated with combined C-H, O-H, and N-H vibrations of lipids, proteins, and amino acids (67).

**Figure 6:**
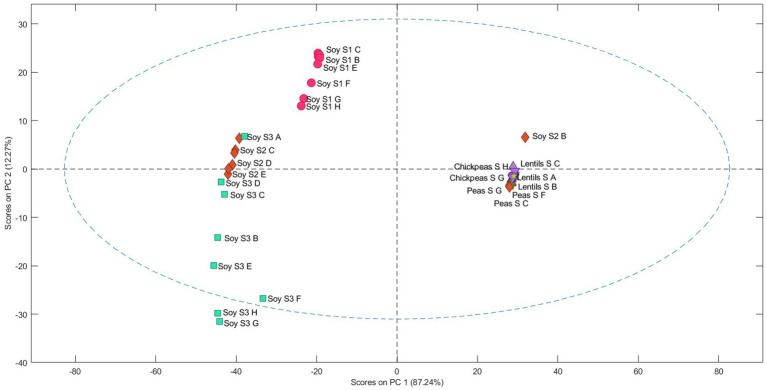
Loadings plot obtained from the PCA model developed using NIR spectral data.

The PCA revealed a clear separation between commercial soy sauces and legume-based condiments (Figure 6). The first principal component (PC1), explaining 87.24% of the total variance, clearly separates soy-based sauces from legume-based samples, indicating that the main source of variability is related to differences in the overall molecular composition of the matrices. Soy sauces are clustered on the negative side of PC1, while pea-, chickpea-, and lentil-based sauces are positioned on the positive side, reflecting distinct compositional fingerprints associated with differences in protein structure, amino acid composition, and carbohydrate profiles.

The second principal component (PC2), explaining 12.27% of the variance, captures variability within the soy-based group, highlighting differences among commercial products. This intra-group dispersion suggests that, in addition to raw material, processing conditions and formulation significantly contribute to the NIR spectral variability. Indeed, NIR spectroscopy is well known to capture not only chemical composition but also physicochemical properties such as water structure, solute concentration, and molecular interactions (64). In this context, the tighter clustering observed for legume-based sauces should be interpreted primarily as a process-related effect rather than solely a consequence of raw material similarity. All legume-based samples were produced by the same manufacturer under the same conditions and using a consistent formulation, which likely minimized variability in physicochemical properties and resulted in highly similar NIR signatures. Conversely, soy-based sauces exhibited a more dispersed distribution, reflecting their origin from different commercial brands characterized by distinct formulations, fermentation conditions, and salt content. Notably, the low-salt soy sauce showed the most distinct positioning within the soy group, indicating a marked compositional deviation. This behavior can be attributed to the strong influence of salt concentration on water activity, osmotic pressure, and molecular interactions within the matrix, all parameters that are effectively captured by NIR spectroscopy. Previous studies have demonstrated that NIR is highly sensitive to key physicochemical properties of soy sauce, including salt content, total solids, density, and osmotic pressure, which are directly linked to processing conditions and formulation strategies (64). Therefore, the separation observed in the PCA reflects not only differences between soy and legume matrices but also the impact of technological variables on the overall molecular organization of the products.

Overall, the NIR-based PCA indicates that both raw material and processing conditions contribute to sample differentiation, with process standardization leading to more compact clustering in legume-based sauces and formulation variability driving dispersion among commercial soy products.

## 4 Conclusion

This study provides an integrated evaluation of commercial soy sauces and innovative legume-based fermented condiments produced using traditional shoyu fermentation techniques. The results clearly demonstrate that substrate composition plays a decisive role in shaping the chemical and sensory profile of fermented sauces. While traditional soy sauces exhibited profiles dominated by fewer high-impact compounds, particularly Maillard-derived heterocycles and volatile phenols, legume-based sauces displayed a broader distribution of volatile compounds, including sulfur-containing metabolites and 1-octen-3-ol, generally associated with mushroom-like aroma. This increased chemical diversity translated into greater aromatic complexity, as confirmed by descriptive sensory analysis. The correlation analysis supports the results obtained from both volatile and sensory profiling. Consumer preference indicated that legume-based sauces were generally preferred over commercial soy sauces, further suggesting that aromatic diversity positively influences hedonic perception. The non-destructive analytical approaches represent complementary tools for identifying these differences. The E-nose effectively captured matrix-dependent variations in global aroma fingerprints, with the majority of sensors strongly associated with legume-based samples. NIR spectroscopy further supported sample differentiation by reflecting differences in overall chemical composition and physicochemical properties. Although the results suggest that substrate composition strongly contributed to sample differentiation, the observed variability also reflects differences in processing conditions, formulation, pasteurization status, and manufacturer-specific practices. Therefore, the present work should be interpreted as a realistic comparison between commercial soy sauces and locally produced soy-free fermented alternatives rather than as a fully controlled substrate-only study. Finally, the study supports the feasibility of developing sustainable, locally sourced fermented sauces with chemical complexity and consumer acceptance comparable to, or in some cases exceeding, those of traditional soy sauces.

## Data Availability

The original contributions presented in the study are included in the article/Supplementary material, further inquiries can be directed to the corresponding authors.
