# Supplementary material for: From soy to legumes: chemical diversity and sensory complexity of fermented sauces revealed by integrated analytical approaches
Source: Front Nutr. 2026 Jun 18;13:1873081. doi: 10.3389/fnut.2026.1873081 (PMC13322842; doi:10.3389/fnut.2026.1873081)
Supplement: Supplementary file 1 [file Table_1.docx]

| Assign a score from **1 to 7 only to the descriptors you perceive.** (1 = Very weak; 7 = Very strong) | |
| --- | --- |
| **Visual** | Color intensity |
|  | Density |
| **Taste** | Sour |
|  | Bitter |
|  | Sweet |
|  | Salty |
|  | Umami |
| **Mouthfeel** | Spicy / Pungent |
|  | Astringent |
|  | Viscosity |
| **Aroma** |  |
| *Fruity* | Black cherry |
|  | Plum |
|  | Cooked fruit |
|  | Dried fruit |
| *Sweety notes* | Honey |
|  | Cotton candy |
|  | Cocoa butter |
| *Spicy* | Clove |
|  | Licorice |
| *Smoky/toasted* | Smoky |
|  | Bacon |
|  | Ash |
|  | Toasted |
|  | Coffee |
|  | Chocolate |
| *Animal/Umami* | Meat |
|  | Leather |
|  | Anchovy sauce |
|  | Shrimp |
|  | Parmesan cheese |
| *Vegetal/Earthy* | Wet soil |
|  | Fresh mushroom |
|  | Dried mushroom |
| *Chemical/Mineral* | Metallic |
|  | Acetic |
|  | Medicinal |
| *Oxidized/Aged* | Marsala wine |
|  | Liqueur-like |
|  | Fermented |
|  | Cellar / Mold |
| *Aftertaste* | Sour |
|  | Bitter |
|  | Sweet |
|  | Salty |
|  | Umami |

**Supplementary table 1**: Sensory sheet used for sensory descriptive analysis of fermented sauces

***Supplementary Material***

**Supplementary Table 2.** Volatiles compounds identified via SPME GC-MS in fermented sauces

| **Chemical Class** | **Identified Compounds** |
| --- | --- |
| **Aldehydes** | Acetaldehyde  Propanal, 2-methyl  2-Hexenal  2,4-Hexadienal (E,E)  Propanal, 3-(methylthio)  Benzaldehyde  Benzeneacetaldehyde  2-Phenyl-2-butenal  Methylbutanal  5-Methyl-2-phenyl-2-hexenal |
| **Ketones** | 2-Propanone  2-Butanone  1-Propanone, 1-(2-furanyl)  Benzyl methyl ketone  Hept-3-yn-2-one  Ethanone, 1-(1H-pyrrol-2-yl)- |
| **Alcohols** | 1-Propanol  1-Propanol, 2-methyl  1-Butanol  1-Butanol, 3-methyl  1-Octen-3-ol  2-Furanmethanol  6-Hepten-1-ol, 5-methyl  Benzyl Alcohol  Phenylethyl Alcohol  1-Phenyl-2-hexyn-1-ol |
| **Esters** | Ethyl Acetate  Pyruvic acid, ethyl ester  2-Furanpropanoic acid, ethyl ester  Lactic acid, ethyl ester  2-Furoic acid, ethyl ester  Benzeneacetic acid, ethyl ester  Acetic acid, phenethyl ester  n-Caproic acid vinyl ester  Pentanoic acid, ethyl ester  Butanedioic acid, diethyl ester  Succinic acid, monomethyl ester  Butanoic acid, ethyl ester  Butyric acid, 2-methyl-, ethyl ester  Ethyl (S)-lactate  Ethyl caproate  Ethyl caprylate |
| **Organic Acids (free)** | 3-(Methylthio)propanoic acid  Propanoic acid, 2,2-dimethyl- |
| **Sulfur Compounds** | 2-Furanmethanethiol  Disulfide, dimethyl  Propanal, 3-(methylthio)-  1-Propanol, 3-(methylthio)-  3-(Methylthio)propanoic acid |
| **Pyrazines** | 2-Ethyl-6-methylpyrazine  Pyrazine, 2-ethyl-5-methyl  Trimethylpyrazine |
| **Phenolic Compounds** | Guaiacol  Ethylguaiacol |
| **Furans / Furanones / Lactones** | Furfural  4-Methyl-5H-furan-2-one  Methyl-γ-butyrolactone  Difurfuryl ether  4-hydroxy-2-methyl-5-ethyl-3(2H)-furanone |
| **Amines** | Ethylenediamine |
| **Polyols / Glycols** | Heptaethylene glycol  3-Hexyne-2,5-diol  Sorbitol (D-Glucitol) |
| **Hydrocarbons / Others** | 2-Methylbutane  2-Methyl-3-methoxy-4H-pyran-4- |

**Supplementary Figure 1.** Scoreplot (PC1 vs PC2) of the PLS-DA built with VOCs identified via SPME GC-MS in fermented sauces


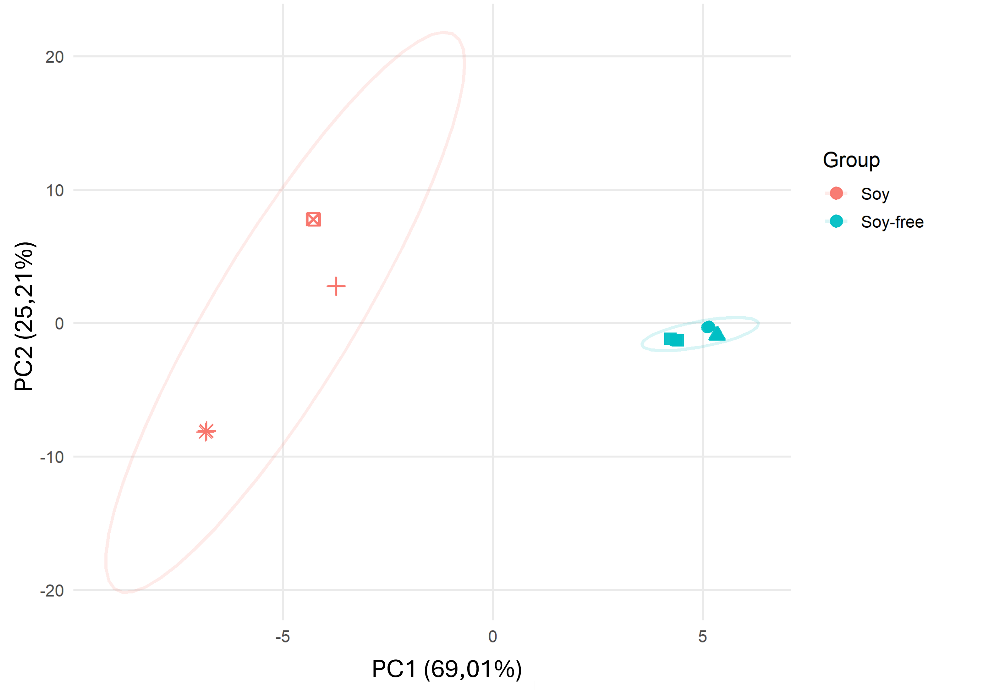


**Supplementary Table 3.** Variable important in projection (VIP score > 0.97) of the PLS-DA built with VOCs identified via SPME GC-MS in fermented sauces

|  | **VIP_PC1** | **VIP_mean12** |
| --- | --- | --- |
| 3-Hexyne-2,5-diol | 1.6440787 | 1.6326315 |
| 2-Furoic acid, ethyl ester | 1.6387903 | 1.6270669 |
| 5-Methyl-2-phenyl-2-hexenal | 1.6348827 | 1.6233798 |
| Ethyl caprylate | 1.6337101 | 1.6220509 |
| D-Glucitol | 1.5593147 | 1.5478998 |
| Disulfide, dimethyl | 1.54747 | 1.5370329 |
| Hept-3-yn-2-one | 1.538468 | 1.5297415 |
| 3-(Methylthio)propanoic acid | 1.4704981 | 1.4598853 |
| Butanedioic  acid, diethyl ester | 1.4615495 | 1.4539259 |
| Sorbitol | 1.4509332 | 1.440424 |
| Ethylguaiacol | 1.4417201 | 1.4323889 |
| Propanal, 2-methyl | 1.4286806 | 1.4182128 |
| Furfuryl alcohol | 1.4199997 | 1.4164492 |
| 2-Furanmethanol | 1.4062182 | 1.4029681 |
| Pyruvic acid, ethyl ester | 1.407634 | 1.3973363 |
| 2-Propanone | 1.3952692 | 1.3849259 |
| 2-Phenyl-2-butenal | 1.239357 | 1.2399426 |
| 1-Propanol, 2-methyl | 1.2300206 | 1.2250208 |
| Guaiacol | 1.1482489 | 1.1496332 |
| 1-Phenyl-2-hexyn-1-ol | 1.122091 | 1.1208923 |
| Heptaethylene glycol | 1.0956281 | 1.0948899 |
| Furfural | 1.0773586 | 1.0775746 |
| 2-Ethyl-6-methylpyrazine | 1.0649289 | 1.0687303 |
| 1-Propanone, 1-(2-furanyl) | 1.0175166 | 1.0190847 |
| 1-Octen-3-ol | 1.0095704 | 1.0110642 |
| Butanoic acid, 2-methyl | 0.9867539 | 0.9895773 |
| HEMF | 0.9899917 | 0.9858552 |
| Lactic acid, ethyl ester | 0.9921232 | 0.9847883 |
| Benzeneacetaldehyde | 0.9804357 | 0.9804998 |
| Ethyl Acetate | 0.9734572 | 0.970272 |
